# Translating translation into patient benefit for atopic eczema

**DOI:** 10.1111/bjd.14909

**Published:** 2016-09-26

**Authors:** N.J. Reynolds, A. Sinha, M.S. Elias, S.J. Meggitt

**Affiliations:** ^1^ Institute of Cellular MedicineNewcastle UniversityNewcastle upon TyneNE2 4HHU.K; ^2^ Department of DermatologyRoyal Victoria InfirmaryNewcastle upon TyneNE1 4LPU.K; ^3^ Present address: Department of DermatologyWye Valley NHS TrustHerefordHR1 2ERU.K

## Abstract

This review considers, in the context of British Skin Foundation (BSF)‐funded translational research into atopic eczema conducted in Newcastle, the complex interactions between clinical and non‐clinical scientists in both academia and industry and how this may have impacted on clinical care. However, research in individual centres does not occur in isolation and clinically relevant outcomes from collaborative research are increasingly supported through regional and national networks. This is illustrated by our trial of azathioprine in adults with atopic eczema conducted across centres in the North East of England that employed pharmacogenetic dosimetry. Correspondingly the formation of a UK Translational Network for Translational Research in Dermatology (UK TREND) has facilitated the development of a UK‐wide network to support atopic eczema projects based on an e‐Delphi prioritisation exercise.

## Abstract 2

**What's already known about this topic?**

- Atopic eczema is a disabling long‐term skin condition that profoundly impacts on patients and their families.
- Ciclosporin remains the only licenced systemic treatment for moderate‐to‐severe atopic eczema in adults.

**What does this study add?**

- Translational research is not linear but represents a complex multi‐disciplinary reciprocal interaction across the clinical/non‐clinical science interface.
- Investigator‐initiated clinical trials in atopic eczema funded by the BSF have influenced national/international guidelines and clinical practice and for the benefit of patients.
- Pharmacogenetic dosimetry for azathioprine is now embedded in dermatological clinical practice.

The Dermatology Research Group in Newcastle places the highest possible priority on the translation of discovery science for the benefit of all users (patients, the NHS, industry, and policy makers) which is in close alignment with the British Skin Foundation's (BSF) strategic aims to further understand the different types of skin disease and to identify better and more effective treatments. We are also strongly committed to increasing public and patient awareness about the impact of disabling skin disease and promoting interaction and education outside of the routine clinical care setting.

In this review, we will consider the outcomes from some of the BSF‐funded research related to atopic eczema conducted in Newcastle and the impact on patient care and future research.

## Evidence‐based treatments for atopic eczema

Atopic eczema is a disabling long‐term skin condition that has a profound negative impact on patients and their families and therapeutic options for moderate‐to‐severe disease remain limited. Although onset is usually in early childhood, atopic eczema may persist (particularly in more severe cases) or recur during adulthood where a prevalence of ~3–10%^1^, ^2^ is reported. A significant percentage of adult patients have resistant disease that can significantly impair quality of life. Refractory moderate‐to‐severe atopic eczema in adults usually runs a prolonged and protracted course^3^ and the unpredictable nature of disease flares is particularly troublesome.^4^ The mainstay of treatment remains topical steroids and moisturisers. In 2000 an independent systematic review^5^ highlighted the lack of therapeutic options for patients with atopic eczema not adequately controlled by optimised topical treatments and underscored the need for scientifically robust testing of a range of treatments for the disease. Indeed, there remains just one oral drug with a product license for moderate‐to‐severe atopic eczema, ciclosporin. Furthermore, the British National Formulary recommends that treatment duration with cicloporin should be limited period for a maximum period of 2 months maximum in part because prolonged use of the drug is associated with hypertension, renal impairment and risk of cancer.^6^, ^7^, ^8^, ^9^ A robust evidence base supporting other treatment options for refractory moderate‐to‐severe atopic eczema was therefore needed.

Following an open pilot study in 2001,^10^ Reynolds and Meggitt, with grant support from the BSF and the Wellcome Trust, led a regional (North East of England) multi‐center placebo‐controlled randomised controlled trial of azathioprine in adult patients with moderate‐to‐severe atopic eczema. This was the first parallel‐group randomised controlled trial of azathioprine for atopic eczema and the first trial for a dermatological condition to utilize a stratified medicine approach in which dosing was tailored to patients based on their genetically‐determined ability to metabolise the drug (as determined by thiopurine methyltransferase [TPMT] activity). The results were published in the Lancet in 2006^11^ and formed the basis of Dr Meggitt's MD thesis (awarded with Distinction). Azathioprine significantly improved disease activity – six area, six sign atopic dermatitis (SASSAD) severity score, the primary endpoint, by 37% (compared to a 20% reduction in the placebo group) as well as body area affected (by 26% compared to 15% by placebo). Importantly, objective improvements in disease activity were matched by improvements in patient‐oriented symptoms and quality of life scores. For example, itch scores reduced significantly (by 46%) in patients who received azathioprine compared to those who received placebo. In 2007, Dr Meggitt also received the British Skin Foundation Award for Best Research Project funded in last 10 years.

The evidence from this study^11^ and a placebo‐controlled cross over trial of azathioprine, also conducted in the UK,^12^ has underpinned recommendations in UK^13^ and European guidelines^14^, ^15^, ^16^ on disease treatment, and azathioprine is now in widespread use in the UK. For example, evidence from a 2013 survey of UK dermatologists conducted through the UK Translational Research Network in Dermatology (UK TREND) and the UK Dermatology Clinical Trials Network (UK DCTN), indicated that azathioprine has been widely adopted in the UK as a treatment of refractory atopic eczema.^17^ Systemic therapy was the most popular second‐line treatment option, chosen by 49% of respondents, and it was also the second most popular first‐line treatment option (after phototherapy), chosen by 36% of respondents. Of those considering systemic therapy, azathioprine was the most popular first‐line choice of drug (46% of respondents). Furthermore, there were clear differences in the pattern of usage and the relative duration of both average and maximum periods of usage between systemic agents. Thus, for example, azathioprine treatment was continued on average, for 13.8 months compared with 5.8 months for ciclosporin – the next most commonly used drug. This may relate to longer‐term control of symptoms and a better safety profile than ciclosporin. In addition, the reported maximum periods of usage indicated that the majority of respondents considered azathioprine as a longer‐term (over 2 year) treatment or maintenance option. Nevertheless, the BAD guidelines on azathioprine advise caution over the prolonged use of azathioprine in part because of uncertainty over cancer risk^13^ and we generally discuss this uncertainty with patients who continue on medication over 12 months.

Based on the reported numbers of patients treated by the consultant dermatologists in the survey, we can estimate that about 4900 patients begin treatment with azathioprine each year in the UK. This compares to ~4450 commencing ciclosporin and ~3950 commencing methotrexate and 7850 being referred for narrow‐band UVB phototherapy. Together this represents a large proportion of the patient pool of adult patients with refractory atopic eczema seen by consultant‐level dermatologists in the UK.

**Figure 1:**
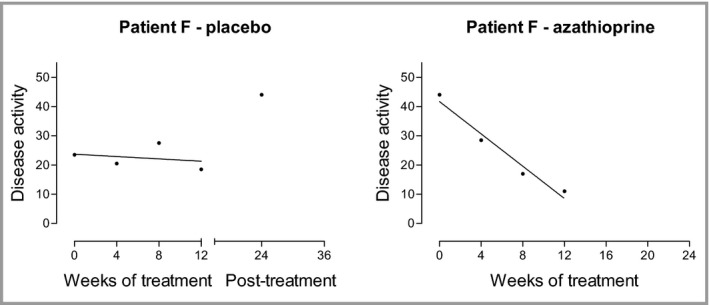
Summary measure of clinical response. Regression lines were fitted to the data points of eczema disease activity (SASSAD) over the 12 weeks of treatment.

**Figure 2:**
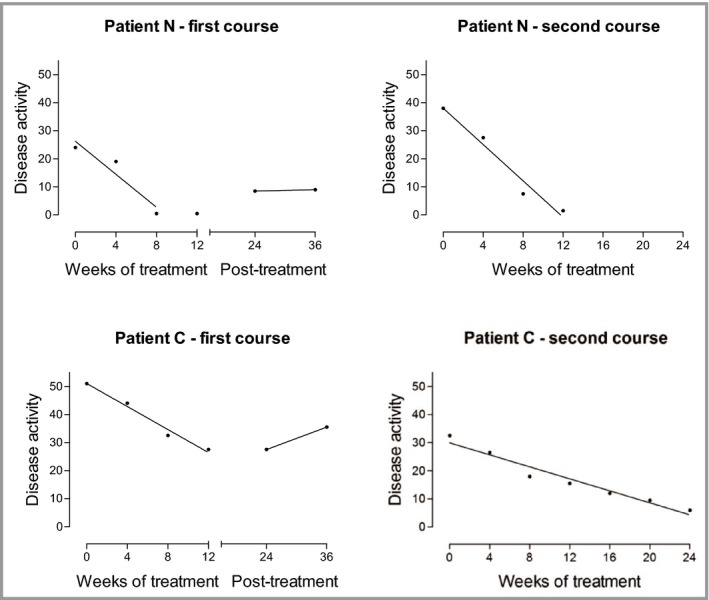
Summary measure of clinical response. Regression lines, fitted to the data points of eczema disease activity (SASSAD) over the treatment period, show a similar slope on repeat courses.

In a previous study in which we compared narrowband UVB phototherapy to UVA phototherapy to placebo,^18^ we had analysed clinical response using a summary measure methodology, as proposed by Matthews.^19^, ^20^ We also applied this methodology to the study of azathioprine in atopic eczema. This method takes into account the variability of disease activity over time and by utilising a series of measurements, during a defined period, provides a more representative summary of response than simply using two measurements at the start and end of a defined period. The variability of baseline measurements is illustrated by patient F (Fig. 1) who was treated with placebo during the trial. The majority of responses were well represented by a linear regression line as shown by patient F (Fig. 1) who later received azathioprine following completion of the study (Meggitt & Reynolds, unpubl. data). Another interesting observation was that in general the pattern of response within individual patients appeared similar upon retreatment as illustrated by patients N and C (Fig. 2).

A full entry on azathioprine appeared for the first time in the skin section of the British National Formulary (a standard resource consulted by UK clinicians when deciding which drug to prescribe) in March 2010. This entry provides further evidence of research impact and states that azathioprine should be considered for severe refractory eczema.

## Pharmacogenetic dosimetry

**Figure 3:**
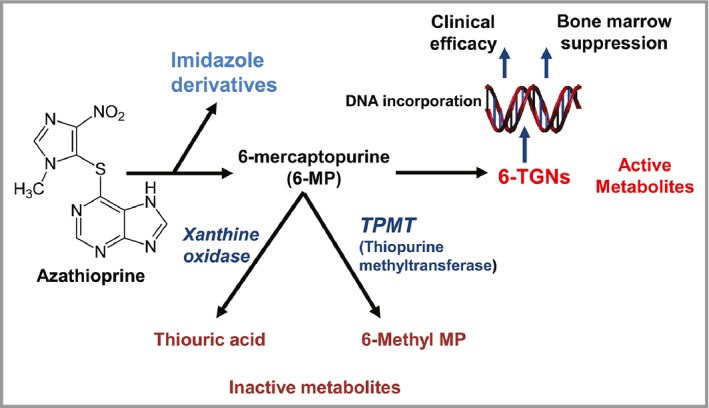
Simplified schematic diagram illustrating metabolism of azathioprine. The pro‐drug azathioprine is converted to 6‐MP with subsequent metabolism to active metabolites 6‐thoiguanine nucleotides (6‐TGNs). Methylation of several thiopurine intermediates by TPMT is important in diverting metabolism away from production of 6‐TGNs. Inhibition of xanthine oxidase by allopurinol may also lead to an increase in 6‐TGNs, hence allopurinol should in general be avoided in patients receiving azathioprine.

As illustrated in Figure 3, azathioprine is a pro drug that is rapidly metabolised to 6‐mercaptopurine (6‐MP). 6‐MP is itself subject to metabolism by several competing pathways that result in a conversion to either inactive metabolites or bioactivation to 6‐thioguanine nucleotides (6‐TGN). 6‐TGNs mediate the therapeutic activity but are also responsible for specific side effects including bone marrow suppression. Thus the balance between enzyme inactivation and activation is critical for optimal therapeutic activity. TPMT metabolises azathioprine (and similar drugs) and genetic variants within the population result in differential ability to break down the drug. Thus, for example, absent TPMT activity (*TPMT* ^*LL*^) may result in high toxic levels of 6‐TGNs and is a contraindication to azathioprine therapy. Further, evidence supports a lower starting dose of azathioprine in patients with intermediate TPMT activity (*TPMT* ^*HL*^). The dosing guidance within the BNF–1–3 mg/kg/day for normal/high TPMT activity (*TPMT* ^*HH*^), 0.5–1 mg/kg/day for low/intermediate TPMT activity (*TPMT* ^*HL*^). –reflects the doses used in our published trial (2.5 and 1 mg/kg/day respectively).

In line with the concept of pharmacogenetic dosing, almost 95% of respondents from our 2013 survey^17^ agreed/strongly agreed that TPMT level at baseline should guide choice of initial dose. These data are consistent with an earlier survey of UK consultant which showed a high level of uptake of TPMT enzyme‐level testing by dermatologists (94%), compared with gastroenterologists (60%) and rheumatologists (47%), prior to prescribing azathioprine.^21^

**Figure 4:**
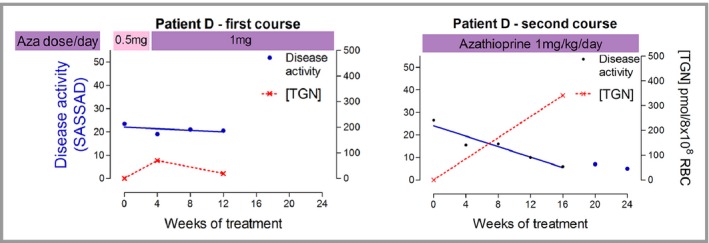
Correlation of clinical response and serum concentrations 6‐thoiguanine nucleotides (6‐TGNs). No clinical response during 1^st^ course with low therapeutic TGN levels but clinical improvement during 2^nd^ course following medication of azathioprine dosage regime and achievement of therapeutic 6‐TGN levels.

A number of studies particularly in inflammatory bowel have demonstrated a correlation between TGN levels and therapeutic efficacy. For example, a therapeutic range of 235–450 pmols/8 × 10^8^ red blood cells has been proposed^22^ and optimization of azathioprine dosimetry on the basis of measuring metabolite levels has led to improved outcomes.^23^ Systematic studies correlating TGN levels with therapeutic efficacy in dermatology are limited although one study has suggested a slightly lower therapeutic threshold of 179 pmol/8 × 10^8^ red blood cells.^24^ However, our unpublished experience in individual cases suggest potential utility with measuring TGN levels particularly in patients with heterozygote TPMT activity. This is illustrated in Figure 4. The therapeutic regime based on TPMT status, as described in the Lancet paper, was used in a TPMT heterozygote patient during the first course of treatment (Fig. 4). Although treatment was well tolerated, the patient showed no therapeutic response and TGN levels remained <70 pmols/8 × 10^8^ red blood cells. Consequently during a second course, the lower dose initiation phase was omitted and TGN levels within the nominal therapeutic range were achieved. This correlated with an improved clinical response (Fig. 4). Of course, such data on individual patients should be interpreted with caution but we are now utilising measurement of TGN levels in clinical practice under such circumstances. On the other hand, a study of 12 children treated with azathioprine showed no obvious correlation between TGN levels and clinical response^25^ although children generally show lower 6‐TGN concentrations compared to adults.^26^

## Mechanism of action of systemic therapies in atopic eczema

Although recent research has provided significant insight into the molecular genetics and pathogenesis of atopic eczema, the mechanism of therapies that are effective in this disease remains incompletely understood. Interestingly, genetic studies provide evidence of distinct genetic loci for atopic eczema and psoriasis and where loci are shared the effect appears to be in the opposite direction for the two diseases.^27^ On the other hand, a number of therapies including topical calcineurin inhibitors, narrow‐band UVB phototherapy and systemic agents such as ciclosporin and methotrexate are effective for both psoriasis and atopic eczema.

## Ciclosporin and cyclophilins

To gain insight into the mechanisms involved in the resolution of atopic eczema we studied the action of ciclosporin as it is one of the most effective systemic agents currently available and has a rapid onset of action. Gaining insight into its mechanism(s) of action in atopic eczema may identify novel therapeutic targets. This would have clinical relevance as ciclosporin usage for atopic eczema is generally limited to short courses due to its potential side‐effects.

Given that the pathogenesis of atopic eczema involves a complex interplay between barrier dysfunction and immune activation and that filaggrin mutations remain the most common genetic risk factor for atopic eczema, we were interested to explore direct effects of ciclosporin and epidermal keratinocytes. The highly conserved family of cyclophilin proteins, characterized by peptidyl‐prolyl cis‐trans isomerase activity, were first identified as binding partners for ciclosporin. Although the interaction between the ciclosporin/cyclophilin A complex, calcineurin and NFAT transcription factors is crucial to inhibition of T cell activation, cyclophilins are ubiquitously expressed. In an earlier study, supported by the BSF, we showed that cyclophilin B (CypB), an endoplasmic reticulum (ER)‐resident protein, is secreted by keratinocytes in response to pharmacologically relevant concentrations of ciclosporin.^28^ Moreover, a key residue within the ciclosporin‐binding site of CypB controlled the secretion of CypB.^28^ These findings may be relevant to the action of ciclosporin in atopic eczema because CypB is known to induce chemotaxis of inflammatory cells and keratinocytes themselves express CypB (CD147) receptors.

To further understand the physiological role of CypB in human keratinocytes, we overexpressed wild type CypB (CypB _WT_) and a mutant protein that is effectively unable to bind ciclosporin (CypB_W128A_). Interestingly, we showed that both CypB _WT_ and CypB_W128A_ increased the proliferative capacity of keratinocytes in both monolayer and in 3‐D skin equivalents.^29^ We also showed that CypB positively regulated keratinocyte differentiation. To understand the relevance of these findings to atopic eczema we studied biopsies taken from lesional atopic eczema skin at baseline and during the early phases of treatment with ciclosporin. Our results show that (1) keratinocyte differentiation was induced early during ciclosporin therapy and (2) CypB expression was modulated in a proportion of subjects. Together these data provide evidence for a direct interaction of ciclosporin with CypB in epidermal keratinocytes which may contribute to the therapeutic effects of ciclosporin in atopic eczema. Further studies to investigate ciclosporin analogues that show relative specificity for CypB appear warranted.

## Methotrexate

Following on from these studies, through a BBSRC‐CASE studentship with Stiefel, GSK we have investigated pathophysiological mechanisms of atopic eczema and the mechanism of action of methotrexate. Interestingly, preliminary results of proteomic studies following knock‐down of filaggrin in epidermal equivalents (to mimic the effects of filaggrin deficiency in atopic eczema skin) identified a number of differentially expressed proteins that included cyclophilin A, further underscoring a potential role for cyclophilins in atopic eczema pathogenesis.^30^ Moreover, our early studies of methotrexate in skin equivalents showed a positive effect on late terminal differentiation in keratinocytes, emphasizing the relevance of the epidermal barrier as a therapeutic target.^31^

## UK TREND

Taking advantage of the collaborative nature of UK dermatology, and building on the success of established networks including BADBIR and the UK DCTN, we have recently established UK TREND. Based on an e‐Delphi prioritisation exercise^32^ and unmet need, UK TREND has focused on developing two networks to support translational research. One of these is centered on atopic eczema. Following two meetings of the atopic eczema network, three sub‐themes emerged that are now being further developed. These are: (1) a longitudinal cohort study of photo and systemic therapy; (2) gene‐environment interactions and (3) stratified medicine. We hope that together the network can take forward research in this area for the future benefit of patients and their families.

In summary, BSF‐funded research in Newcastle has provided insight into pathophysiological mechanisms in atopic eczema, as well as the pharmacology and pharmacogenetics of systemic therapeutic agents. These findings have been translated into improved patient care. In part through the support of individual research careers and support nationally for organisations such as UK TREND, we are now poised to further advance understanding of atopic eczema with the real prospect of personalized therapeutics in this disabling long‐term condition.
